# Trait Dominance Promotes Reflexive Staring at Masked Angry Body Postures

**DOI:** 10.1371/journal.pone.0116232

**Published:** 2014-12-30

**Authors:** Ruud Hortensius, Jack van Honk, Beatrice de Gelder, David Terburg

**Affiliations:** 1 Cognitive and Affective Neuroscience Laboratory, Tilburg University, Tilburg, The Netherlands; 2 Department of Experimental Psychology, Utrecht University, Utrecht, The Netherlands; 3 Department of Psychiatry and Mental Health, University of Cape Town, Cape Town, South Africa; 4 Institute of Infectious Diseases and Molecular Medicine, University of Cape Town, Cape Town, South Africa; 5 Brain and Emotion Laboratory, Department of Cognitive Neuroscience, Faculty of Psychology and Neuroscience, Maastricht University, Maastricht, The Netherlands; 6 Brain and Emotion Laboratory Leuven, Division of Psychiatry, Department of Neurosciences, KU Leuven, Leuven Belgium; University of Bologna, Italy

## Abstract

It has been shown that dominant individuals sustain eye-contact when non-consciously confronted with angry faces, suggesting reflexive mechanisms underlying dominance behaviors. However, dominance and submission can be conveyed and provoked by means of not only facial but also bodily features. So far few studies have investigated the interplay of body postures with personality traits and behavior, despite the biological relevance and ecological validity of these postures. Here we investigate whether non-conscious exposure to bodily expressions of anger evokes reflex-like dominance behavior. In an interactive eye-tracking experiment thirty-two participants completed three social dominance tasks with angry, happy and neutral facial, bodily and face and body compound expressions that were masked from consciousness. We confirmed our predictions of slower gaze-aversion from both non-conscious bodily and compound expressions of anger compared to happiness in high dominant individuals. Results from a follow-up experiment suggest that the dominance behavior triggered by exposure to bodily anger occurs with basic detection of the category, but not recognition of the emotional content. Together these results suggest that dominant staring behavior is reflexively driven by non-conscious perception of the emotional content and triggered by not only facial but also bodily expression of anger.

## Introduction

*“A proud man exhibits his sense of superiority over others by holding his head and body erect. He is haughty (haut), or high, and makes himself appear as large as possible; so that metaphorically he is said to be swollen or puffed up with pride.”* [1], p. 142.

Social dominance is often established and maintained through direct gaze and sustained eye-contact. The mechanism underlying such staring-contest behavior is fundamental to the establishment of social hierarchies and is found in humans and other primates [2], [3]. Dominance and submission are, however, not exclusively conveyed or provoked through facial features. One only has to imagine the figure of an approaching person in a dark alley to appreciate that body language might be an important factor in dominance-submission interactions. Indeed, briefly adopting a high-power pose may lead to dominance-related changes such as increased testosterone and decreased cortisol levels, heightened risk-taking, and increased feelings of power [4]. In the observer, the perception of a threatening bodily expression can subsequently trigger neural mechanisms underlying automatic defensive action [5], [6].

The relation between sustained eye-contact and personality traits of dominance resembles a non-conscious reflex-like mechanism [3], [7]. Despite the ecological validity and biological relevance of body postures [8], surprisingly few studies have been conducted on their interplay with personality traits and behavior. Here we aim to fill this gap by investigating whether angry bodily expressions that are not perceived consciously evoke reflex-like dominance behavior similar to the staring-contest as was shown previously with slower gaze-aversion from angry compared to happy faces [3], [7]. Given the strong link between aggression and dominance [2], and the notion that physical aggression is acted out with the body, we expect that these gaze-aversion effects will generalize to bodily dominance-cues.

## Social Dominance Experiment

### Methods

#### Ethics Statement

The research reported in this article involves healthy human participants, and does not utilize any invasive techniques, substance administration or psychological manipulations. Therefore, compliant with Dutch law, this study only required, and received approval from our internal faculty board (Human Biopsychology and Psychopharmacology) at Utrecht University. Furthermore, written informed consent of each participant was obtained and this research was conducted according to the principles expressed in the Declaration of Helsinki.

#### Participants

Thirty-two healthy individuals (sixteen females), aged between 19 and 26 years, participated in exchange for course credit or eight Euros. Participants were unaware of the aim of the study.

#### Stimuli and tasks

The same angry, happy and neutral facial expressions (five male, five female actors) from [9] were used as in Terburg et al. [3], [7]. A mask was made from cut-up and randomly reassembled faces. Angry, happy and neutral bodily expressions (five male, five female actors) were taken from the Tilburg Stimulus Set [10]. The neutral control expression was an instrumental action (cf. making a telephone call). All three expressions were well recognized in a separate group of students (*n* = 24; mean±sd percentage correct for angry: 91.30±2.29, happy: 98.26±0.81, neutral: 97.39±1.57). In addition to the isolated facial and bodily expressions, we tested if the effects were generalizable to full emotional expressions including facial and bodily signals. Therefore we constructed face-body compounds [11] by combining these expressions (see **S1 Fig.**). Using Photoshop CS2 (Adobe Systems Inc., San Jose, CA, USA) faces from the MacBrain Face Stimulus Set were carefully resized and positioned on top of the body using realistic proportions (face-body ratio of 1∶7). Contrast and brightness of the face was adjusted to match the values of the body. Only congruent compounds were created (e.g. angry face with angry body). The mask for bodily and compound expressions consisted of a scrambled image of all stimuli combined.

We used a procedure similar to Terburg and colleagues [3]. Participants performed three eye-tracking tasks with faces, bodies or face-body compounds as target stimuli. Face-only and body-only tasks were tested first (counterbalanced across participants). The compound task was presented last to prevent the more complex stimuli from interfering with their simpler counterparts due to repetition effects. In each trial a gray pre-mask with a central fixation-cross (random interval between 1000–1500 ms) preceded a red, green or blue emotional target-stimulus (angry, happy or neutral), followed by a post-mask of similar luminance and color. In order to prevent habituation to the masking, different versions of the masks were used. We selected a target presentation time of 14 ms [12], because the previously reported effects of trait dominance on gaze-aversion were observed exclusively in individuals that were fully unaware of the masked facial emotions [3] and bodily expressions are confidently detectable at presentation durations of 33 ms [13].

**Figure 1:**
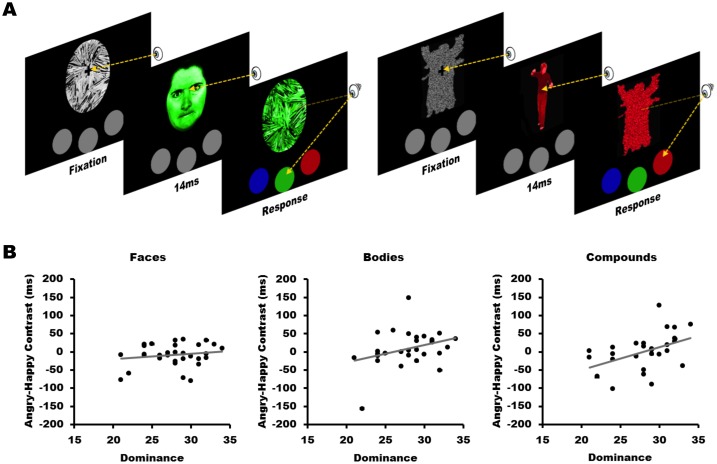
Illustration of the social dominance task and results. **A.** Outline of the social dominance task (figure adapted from [3]). **B.** Dominance increases gaze duration to angry bodily and compound expressions, but not to facial expressions.

Participants’ task was to avert gaze as fast as possible to one of three circles below the stimulus with the same color (see **Fig. 1A**). The emotional expressions were presented in a fixed sequence, repeated five times (NxxyNyyxNNyyxNxxyN; N = neutral; x and y = angry or happy counterbalanced over participants), in order to ensure that all successive trial-types occurred equally often [7]. Before the onset of each task, participants performed 10 neutral practice trials. Stimuli were presented on a 17-inch CRT monitor. The session was concluded with three 30-trial awareness checks, with the stimuli presented in the same manner as the social dominance task, but with the instruction to identify the emotion of the masked target in a 3-alternative-forced choice design (3AFC).

#### Trait dominance

Participants completed the Behavioral Activation Scale (BAS) [14], as a measure of trait dominance and non-dominance related reward sensitivity. The BAS questionnaire consists of three subscales: fun-seeking (BASF; e.g., “I will often do things for no other reason than that they might be fun”), drive (BASD; e.g. “I go out of my way to get things I want”), and reward responsiveness (BASR; e.g. “It would excite me to win a contest”). These subscales have successfully been used to distinguish between dominance (BASD and BASR) and non-dominance related reward sensitivity (BASF) [3], [14].

#### Data analysis

Gaze latencies (time between target onset and first gaze on target-circle) were recorded with a Tobii X120 binocular eyetracker sampling at 120 Hz (Tobii Technology, Danderyd, Sweden). Latencies shorter than 100 ms or more than 3SDs from the individual’s mean within each task were discarded, and mean latency was computed for each emotional condition in each task, and used for further analysis.

Dominance-related BAS scores were calculated by combining the scores on the drive and reward-responsiveness BAS scale, *r_s_*(32) = .67, *p*<.001 [3]. Non-dominance related BAS scores were defined as the score on the fun-seeking BAS scale. Dominance and non-dominance related BAS scores were not significantly related, *r_s_*(32) = .13, *p* = .48.

Individuals who scored significantly above chance-level (>14 correct; chance level = 10 correct on 30 trials; binomial test with one-tailed *α* = .05) on the objective awareness-check were excluded from further analyses (face: null, body: three, compound: five). Using a general linear model (GLM) for repeated measurements, we tested for each task separately if emotional expression influenced gaze duration. In line with previous studies [3], [7], linear regression analyses were used for the three tasks separately on the angry-happy contrast with dominance and non-dominance related BAS-scores as predictor variables.

## Results

No main effect of emotion was found for facial, *F*(2,62) = 1.00, *p* = .37, bodily, *F*(2,56) = 1.31, *p* = .28, or compound expressions, *F*(2,52) = 0.02, *p* = .98. Significant regression models were observed for bodily, *F*(2,26) = 9.16, *p* = .001, *R^2^* = .41, and compound, *F*(2,24) = 3.47, *p* = .05, *R^2^* = .22, but not for facial, *F*(2,29) = 1.11, *p* = .35, *R^2^* = .07, expressions. Consistent with our predictions, slower gaze-aversion from angry compared to happy bodily expressions was positively related to dominance traits (β = .48, *p* = .005) and negatively to non-dominance related reward sensitivity (β = −.57, *p* = .001; see **Fig. 1B**). These results were similar when two individuals with bias scores >±150 ms were removed, *F*(2,24) = 9.39, *p* = .001, R^2^ = .44, with dominance traits (β = .40, *p* = .02) and non-dominance related reward sensitivity (β = −.65, *p*<.001) as predictors. Dominance traits also positively predicted gaze-aversion from angry compared to happy compound expressions (β = .44, *p* = .02), but non-dominance related reward sensitivity did not contribute significantly to this model (β = .15, *p* = .40).

## Discussion

As hypothesized, we observed slower gaze-aversion from non-conscious angry compared to happy body postures in relation to dominance traits. This effect was similar for body and compound stimuli and is in line with previous studies using face stimuli presented at a longer stimulus duration [3], [7]. This suggests a robust effect of body-evoked dominance behavior. However, in the present study we did not observe the same effect with face stimuli. Importantly, the present and previous [3] study were similar except for presentation duration of the target stimuli. In the previous study the faces were presented for 33 ms and the dominance effect was exclusively found in the participants, about two-thirds of the sample, that were fully unaware of the emotional content of the stimuli [3]. Crucially, although faces (but in general not their emotional expression) are detectable at 33 ms, they are fully undetectable at 14 ms [15]. Body postures, but again not their emotional expression, might have been detected at such short durations. Therefore, we cannot exclude that the reflexive dominance behavior we observe in our experiments depends on some form of basic detection of the stimuli. We therefore tested the hypothesis that at 14 ms presentation duration bodies are detectable, but faces are not.

## Control Experiments

### Methods

#### Participants

Twenty healthy individuals (ten females) aged between 18 and 24 years participated in exchange for course credit. The participants did not take part in the social dominance experiment and were unaware of the aim of the study.

#### Stimuli and tasks

Participants performed eight short experiments in which they had to detect the occurrence of a target-stimulus (detection task) or recognize the target-emotion (emotion recognition task). We used four different stimulus durations (10/14/20/28 ms). Refresh rate of the CRT monitor was adjusted with respect to the duration of the stimulus (i.e. for a stimulus duration of 10 and 20 ms the refresh rate was changed to 100 Hz). Duration and target-stimulus were counterbalanced across participants. The same stimuli and trial procedure were used as in the social dominance task. Either faces or bodies served as target-stimuli. In each trial a gray pre-mask preceded a colored target-stimulus (happy, angry, or neutral expression), which was followed by a post-mask of similar color, shown until response. In the detection task participants indicated if they had seen the target-stimulus (yes/no), while in the emotion recognition task the participants indicated the emotion. In the detection task 50% of the trials contained no stimulus. For each condition twelve trials were shown, with a total of 576 trials in the detection task and 288 trials in the emotion recognition task.

#### Data analysis

For the detection task we calculated the d-prime (d’), which measures the distance between signal and noise [16]. With a d’ of 0 the individual cannot discriminate between signal and noise, whereas a d’ of 1 suggests medium performance and a d’ of 4.65 suggests optimal performance. The d’ is calculated with the following formula:



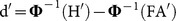



We used the formula proposed by Snodgrass and Corwin [17] to calculate corrected hit rate (H’) and corrected false alarm rate (FA’) out of the hits (h), correct rejections (cr), misses (m) and false alarms (f):



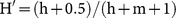





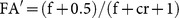



To test differences in detection (d’) between facial and bodily expressions at different stimulus durations, a general linear model (GLM) for repeated measurements with stimulus-type (2) and duration (4) as within subject factors was used. A similar approach was used for emotion recognition (number of trials correct). In addition, we tested if emotion recognition for each target-stimulus was significantly different from chance level at each duration (36 trials in total per target-stimulus per duration, chance level = 12) by means of one sample t-test. Post-hoc paired samples t tests were Bonferroni-corrected.

## Results

### 

#### Detection

A main effect of type of stimulus was found, *F*(1,19) = 17.82, *p*<.001, η_p_^2^ = 0.48. Post-hoc t-tests showed that the d’ for bodies was significantly higher compared to faces at all durations (*p*’s≤.01). Furthermore, the d’ for bodies was significantly different from zero at all durations (*p*’s≤.008), whereas the d’ for faces was only significant from zero with a duration of 28 ms (*p* = .04). A main effect of duration, *F*(3,57) = 6.15, *p* = .006, η_p_^2^ = 0.25 was observed. The overall d’ at 28 ms was significantly higher compared to 14 ms, *t*(19) = −3.42, *p* = .02. No significant interaction between type of stimulus and duration was observed, *F*(3,57) = 0.06, *p* = .98. See **Table 1** for d’ values across conditions.

**Table 1:** Results for detection and emotional recognition tasks.

	10 ms	14 ms	20 ms	28 ms
*Detection*				
Faces	−0.03±0.06	0.04±0.05	0.16±0.08	0.43±0.15
Bodies	0.98±0.25	1.02±0.28	1.22±0.28	1.47±0.37
*Emotion recognition*				
Faces	11.90±0.43	11.70±0.42	11.45±0.44	11.75±0.50
Bodies	13.45±0.89	14.15±0.80	14.00±1.03	14.45±1.20

Mean ± standard error d’ reported for detection; mean ± standard error number of trials correct reported for emotion recognition.

#### Emotion recognition

Number of trials correct differed between type of stimulus, *F*(1,19) = 5.70, *p* = .03, η_p_^2^ = 0.23. Participants had more trials correct when recognizing bodily (14.01±0.87) compared to facial (11.70±0.26) expressions. Importantly, for both target-stimuli the number of trials correct at each duration was not significantly different from chance-level (12 correct; *p*’s>.22), except for a marginally significant difference for bodies presented at a duration of 14 ms (*p* = .06). No main effect of duration was observed, *F*(3,57) = 0.26, *p* = .85. Furthermore, no significant interaction between type of stimulus and duration was found, *F*(3, 57) = 0.69, *p* = .56. See **Table 1** for number of trials correct across conditions.

## Discussion

As expected, with none of the presentation durations were the participants able to recognize the emotional expression of the masked faces or bodies. In contrast, stimulus detection performance was different for faces and bodies. Results showed medium performance for body detection, but detection of faces was only significantly different from zero at a presentation time of 28 ms. The latter result suggests that the faces in the social dominance experiment remained undetected. Moreover, although their emotional expressions were successfully masked, the bodies in the social dominance experiment, as well as the faces in our previous experiments [3], [7], were most likely detectable.

## General Discussion

In the present study we investigated whether dominant individuals exhibit reflex-like gaze behavior when confronted with bodily anger. In support of our hypothesis we show for both bodies, and compounds, a positive relationship between trait dominance and slower gaze-aversion from non-consciously processed angry compared to happy expressions. The results from the control experiments suggest that the absence of gaze-aversion effects with facial expressions in the present experiment may be related to the fact that faces, but not bodies, are undetectable at presentation times of 14 ms. It is important to note that in the social dominance task using bodies or faces, the stimulus property that varies and therefore needs to be masked is the emotional expression [18]. Given that emotional expressions were successfully masked in the present as well as in previous studies using this task [3], [7], the results point at non-conscious effects of facial (previous study) and bodily (present study) anger on dominance behavior, that is, in the absence of critical awareness of the emotional content [18].

Bodily expressions signal intentions and actions, and have been suggested to automatically trigger action responses [8]. They activate subcortical mechanisms [5], [6] associated with early emotional processing and reflexive action [19]. Recent evidence on the combination of dominance traits, electrophysiology, endocrine functions and behavioral responses to facial anger suggests that staring-behavior for dominance is rooted in a relatively increased subcortical over cortical processing mode [20], and mediated by the steroid hormone testosterone [7] (see [21] for a review). Involvement of testosterone in staring-contests has also been suggested in other primate species [2], which underscores the importance and adaptive relevance of this type of dominance behavior [1]. As such, these results provide for the first behavioral evidence that non-conscious bodily anger can evoke ecologically valid, reflex-like dominance behavior.

Interestingly, although we did not observe dominance behavior in relation to facial anger, behavioral effects using the same threshold (14 ms) have previously been found when using fearful faces [12]. This intriguing difference might reflect the evolutionary relevance of fear over anger as a signal of predatory danger [22], but further research is needed to substantiate this claim [23]. In addition, bodily expressions of anger might bias perception towards adaptive action (‘I need to dodge the punch’) whereas facial expressions of anger might bias perception towards understanding intention (‘why is the person angry at me?’) [8]. Notwithstanding that angry facial expression still trigger reflexive behavior with longer stimulus duration [3], bodily signals of threat might simply be more effective in triggering dominance behavior.

The present and previous results [3] suggest that basic detection, but not recognition, of the emotional content, lies at the foundation of the relation between trait dominance and reflexive staring. Detection and recognition of bodily expressions, but also facial expressions, are possibly mediated by distinct but connected parallel neural routes with different behavioral outcomes [19], [24]. It is important to note that detection and recognition are not necessarily dependent upon each other, i.e. detection can occur without recognition and vice versa. Indeed, a recent study showed that above chance-level emotion categorization of a facial expression may take place when observers cannot reliably categorize the stimulus as either a face or an object [25]. An interesting phenomenon in this respect is ‘affective blindsight’ (AB), which describes patients with cortical blindness who can still process some of the emotional content of visual information [26]–[32]. It is thought that AB is driven by subcortical brain regions, such as the amygdala, pulvinar, and superior colliculus [33], [34], which is in line with the above proposed involvement of subcortical areas in reflexive dominance behavior. Furthermore, in line with our results AB patients seem to be able to detect bodies, but not faces, above chance-level [30], whereas the emotional content of both faces and bodies do evoke affective responses [32]. Importantly, these effects are non-conscious in nature and as such might resemble the non-conscious emotional modulation of dominance behavior observed in the present study.

In conclusion, the present study replicates and extends previous research on dominance behavior [3], [35] and provides important insights into reflexive social behavior. Exposure to angry bodily expressions can drive reflex-like gaze behavior and this finding provides a new window on the interplay between personality traits and behavioral reflexes.

## Supporting Information

S1 Data **Data of the social dominance experiment.**
(XLSX)

S1 Fig **Examples of compound expressions.**
(TIF)

S2 Data **Data of the control experiments.**
(XLSX)
