# Supplementary figures and images for: Trait Dominance Promotes Reflexive Staring at Masked Angry Body Postures
Source: PLoS One. 2014 Dec 30;9(12):e116232. doi: 10.1371/journal.pone.0116232 (PMC4280224; doi:10.1371/journal.pone.0116232)

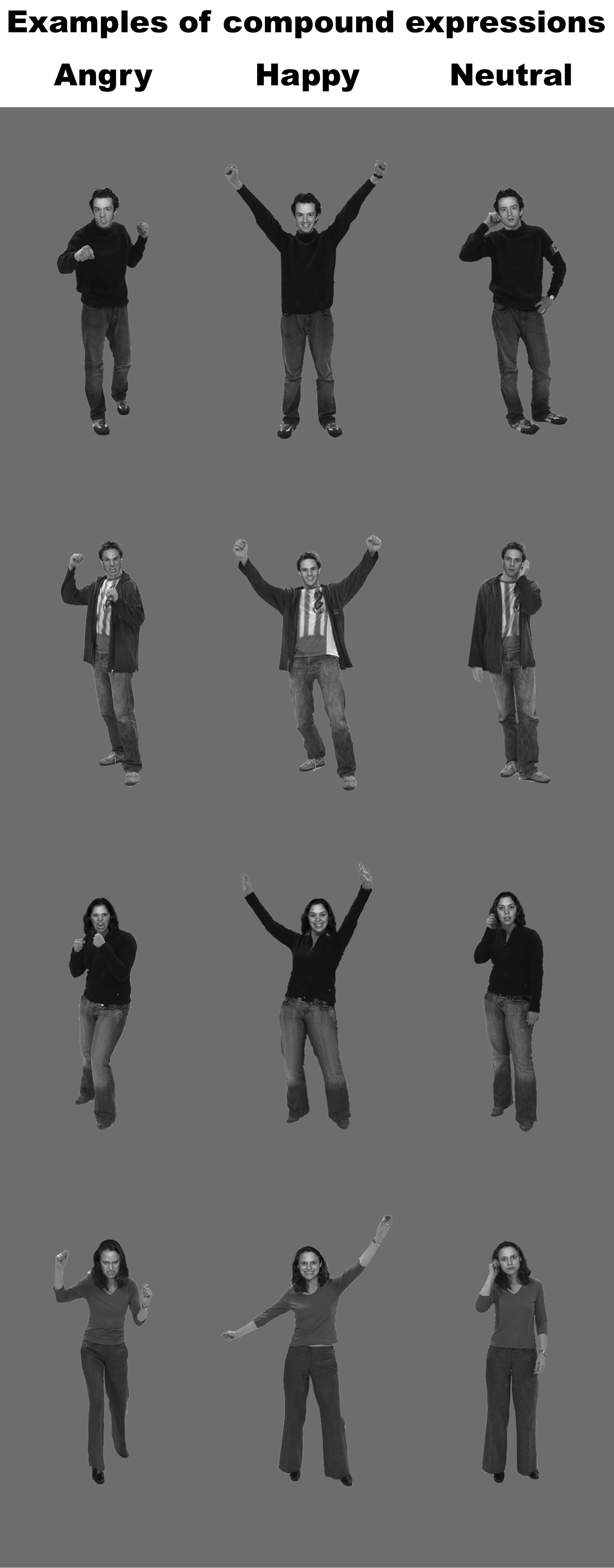

Supplement: S1 Fig — Examples of compound expressions. (TIF) [file pone.0116232.s002.tif]
